# Screening of young competitive athletes for the prevention of sudden cardiac death with a wireless electrocardiographic transmission device: a pilot study

**DOI:** 10.1186/s13104-015-1311-9

**Published:** 2015-08-11

**Authors:** Jae Hyung Cho, Mats A Selen, Abraham G Kocheril

**Affiliations:** Department of Hospital Medicine, Cleveland Clinic, Cleveland, OH USA; Department of Physics, University of Illinois at Urbana-Champaign, Champaign, IL USA; Department of Cardiology, College of Medicine, University of Illinois at Urbana-Champaign, 101 West University Avenue, Champaign, IL 61820 USA

**Keywords:** Electrocardiogram, Screening, Sudden cardiac death, Athletes

## Abstract

**Background:**

The 12-lead electrocardiographic screening for the prevention of sudden cardiac death in young competitive athletes is not cost-effective and thus not routinely recommended. We investigate whether a less expensive wireless electrocardiographic transmission device can be used to screen for the prevention of sudden cardiac death in this population.

**Methods:**

During pre-participation screening, twenty college football players underwent two electrocardiograms: a conventional 12-lead electrocardiogram and a wireless 9-lead electrocardiogram. We compared several electrocardiographic parameters (QRS duration, left ventricular hypertrophy using the Cornell voltage criteria and the Sokolow–Lyon criteria, ST deviation and corrected QT interval) to determine the correlation.

**Results:**

The QRS duration, left ventricular hypertrophy using the Cornell voltage criteria and the Sokolow–Lyon criteria and corrected QT interval exhibited significant correlation between the two types of electrocardiograms (correlation coefficient 0.878, 0.630, 0.770 and 0.847, respectively with P values of 0.01, 0.003, 0.01 and 0.01, respectively). ST deviation in V1 was weakly correlated between the two types of electrocardiograms without statistical significance (correlation coefficient 0.360 with a P value of 0.119).

**Conclusions:**

Our newly developed wireless 9-lead electrocardiogram demonstrated significant correlations with a conventional 12-lead electrocardiogram in terms of QRS duration, left ventricular hypertrophy and corrected QT interval.

## Background

Sudden cardiac death among young competitive athletes was reported occurring in 0.46 per 100,000 athletes per academic year in high school grade 10–12 in Minnesota [1]. In the United States, the most common cause of sudden cardiac death among athletes has been reported as hypertrophic cardiomyopathy (26.4 %) [2]. An observational study in Italy showed that sudden cardiac death in young athletes aged 12–35 years occurred in 2.3 athletes per 100,000 per year [3]. Arrhythmogenic right ventricular cardiomyopathy was the most common cause (22.4 %) of sudden cardiac death in Italian athletes [4].

The 12-lead electrocardiogram (ECG) for the screening of sudden cardiac death in young competitive athletes is recommended by the European Society of Cardiology (ESC), but not by the American Heart Association (AHA) and American College of Cardiology (ACC) [4, 5]. The ESC recommended mandatory 12-lead ECG for sudden cardiac death screening in young competitive athletes based on the Italian study conducted by Corrado et al. [6], which showed a 79 % relative risk reduction attributed to screening. In Europe, the cost of a 12-lead ECG is roughly 10 Euros (1 Euro is approximately 1.36 dollars as of January 2014) [4]. In the United States, however, the cost of a 12-lead ECG is expected to be 39–47 Dollars according to the Medicare and Outpatient Prospective Payment Systems institutional reimbursement rates [7]. A cost-projection model showed that the cost per life saved would be between 10.6 and 14.4 million Dollars in the United States [7].

A less expensive wireless electrocardiographic transmission device could make screening feasible for the prevention of sudden cardiac death. A new 9-lead wireless electrocardiographic transmission device was developed by a physicist at the University of Illinois at Urbana-Champaign. We investigated whether this new 9-lead ECG could be used to screen for the prevention of sudden cardiac death in young competitive athletes.

## Methods

### Screening population

Football players at the University of Illinois at Urbana-Champaign were enrolled for the pre-participation screening in our study. We held a meeting with freshman football athletes in the absence of their coaches during the pre-participation screening for the 2013–2014 season. We explained our study objectives, protocols and benefits and answered their concerns and questions. Written informed consent was obtained from each athlete. Twenty freshman college football players at the University of Illinois at Urbana-Champaign were enrolled in our pilot study. They were free to withdraw from our study at anytime.

### New 9-lead wireless electrocardiographic transmission device

**Fig. 1:**
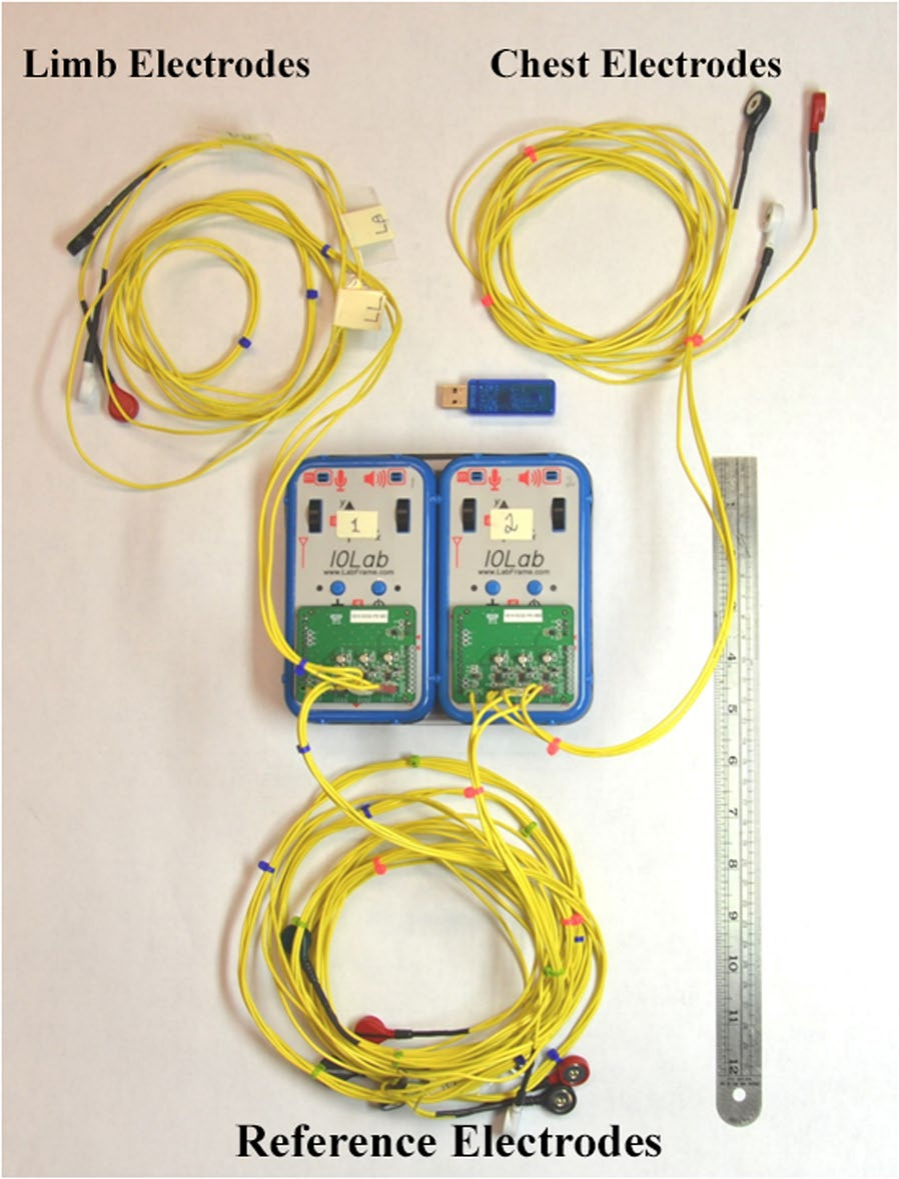
The new wireless 9-lead electrocardiographic transmission device.

**Fig. 2:**
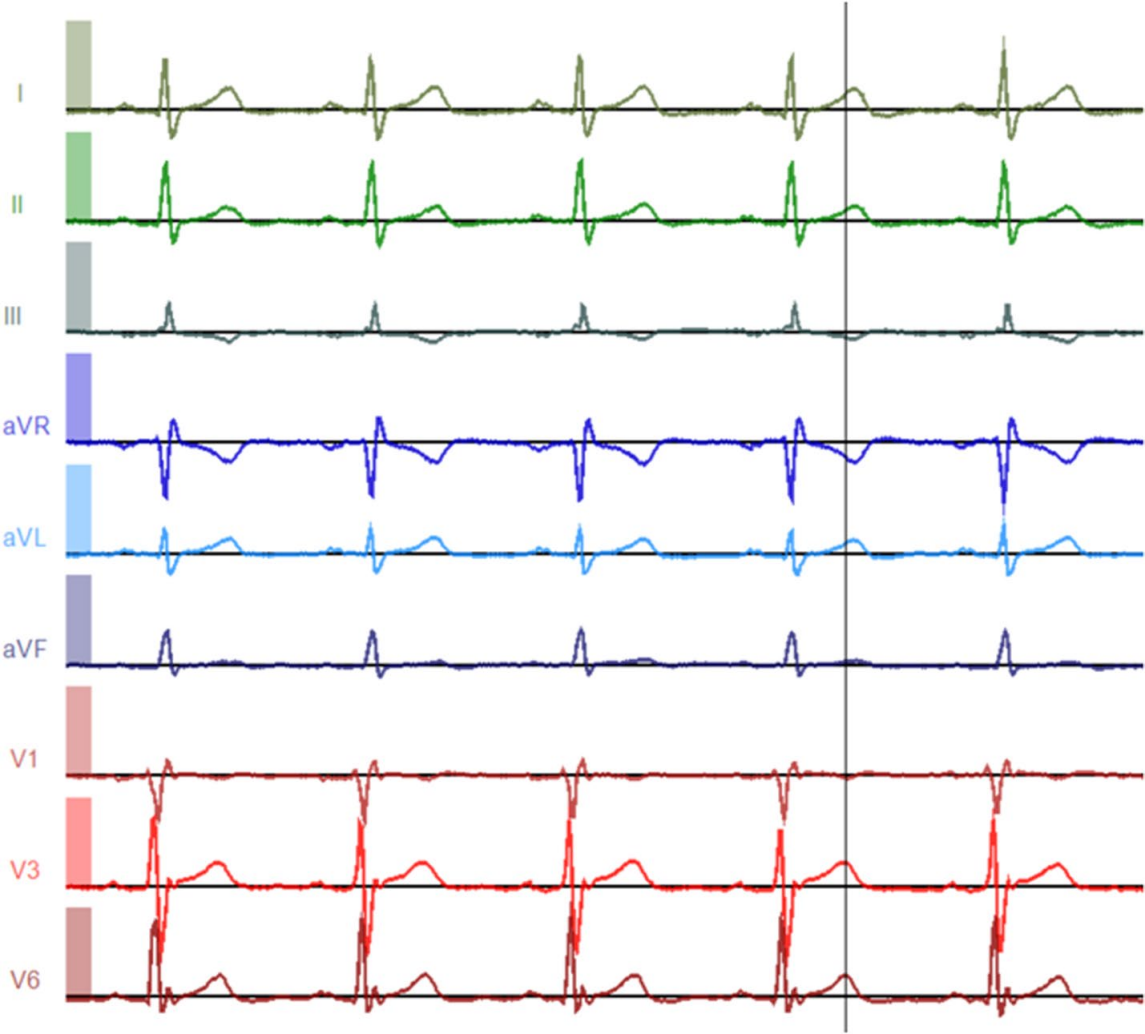
A 9-lead ECG obtained from the new electrocardiographic device.

**Fig. 3:**
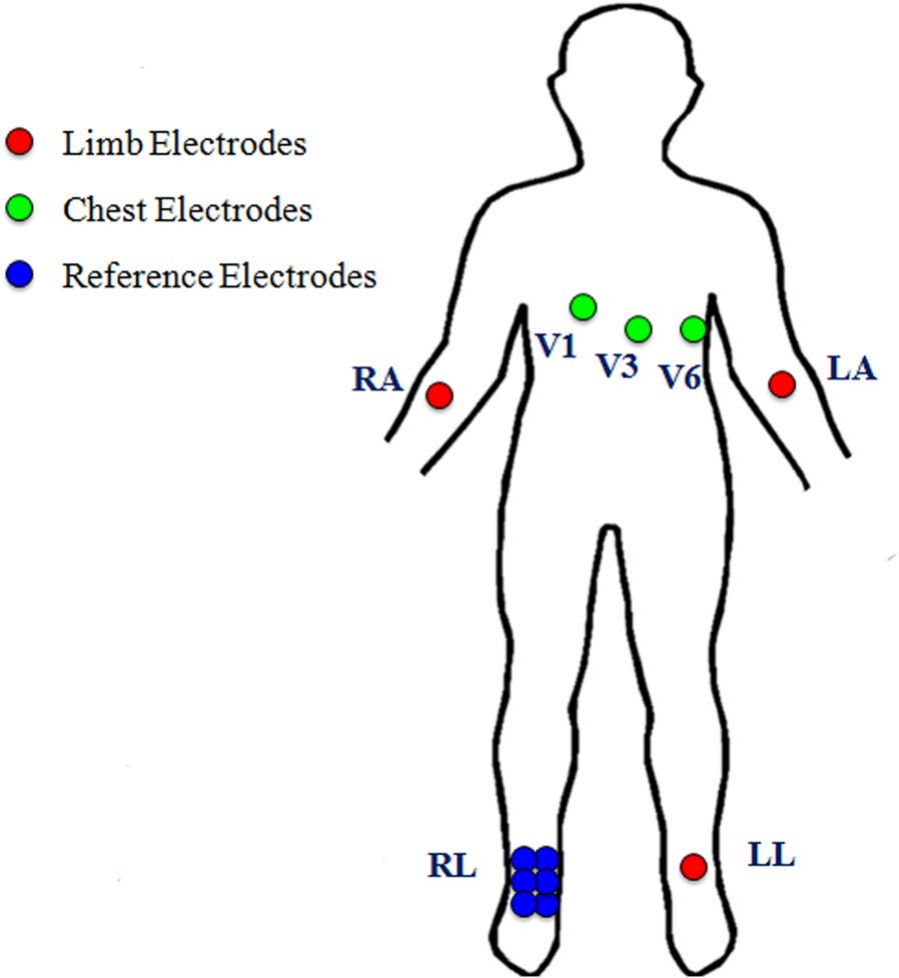
Lead placement for the new 9-lead electrocardiographic recording.

A physicist at the University of Illinois at Urbana-Champaign has invented a new 9-lead wireless electrocardiographic transmission device (Fig. 1). The electrocardiogram produced by this device is obtained using 9 leads, lead I, II, III, aVR, aVL, aVF, V1, V3 and V6 (Fig. 2). The device utilizes 9 leads similar to the conventional 12-lead ECG, omitting lead V2, V4 and V5. There are a total of 12 electrodes, 3 of them limb electrodes, 3 of them chest electrodes and 6 of them reference electrodes (Fig. 3). The IOLab device was designed to be an inexpensive wireless transmission device for teaching, but its low cost, coupled with its battery operation and flexible expansion port, makes it ideal as a low-noise electrical data-recording device.

### Screening process

During pre-participation screening for the 2013–2014 season, 20 freshman college football players at the University of Illinois at Urbana-Champaign underwent two ECGs. Each athlete underwent a conventional 12-lead ECG screening, followed by the new 9-lead ECG screening on the same day. The results from the conventional 12-lead ECGs were printed and numbered and the results from the 9-lead ECGs were wirelessly transmitted to a secure server and numbered the same as the respective 12-lead ECGs.

### Electrocardiographic parameters

We compared several electrocardiographic parameters between the two ECGs. To screen for pre-excitation syndrome, arrhythmogenic right ventricular cardiomyopathy and bundle branch blocks, we measured and compared the QRS durations between the two ECGs. For hypertrophic cardiomyopathy and left ventricular hypertrophy (LVH), two criteria were used: the Cornell voltage criteria (R wave in lead aVL + S wave in lead V3 >2.8 mV) and the Sokolow–Lyon criteria (S wave in lead V1 + R wave in lead V6 >3.5 mV). To screen for Brugada syndrome and arrhythmogenic right ventricular cardiomyopathy, the ST deviation in lead V1 was measured and compared. For the screening of long QT and short QT syndrome, the corrected QT intervals were checked. The electrocardiographic parameters were measured in a blinded manner using serial numbers. Calipers were used to measure the electrocardiographic parameters on the 12-lead ECG and a digital ruler was utilized to measure the electrocardiographic parameters on the 9-lead ECG. The abnormal findings of 12-lead ECGs for the athletes are summarized in Table 1.

**Table 1:** Abnormal 12-lead ECG findings of the twenty athletes

Abnormal findings	Athlete number	Detail
QRS duration >120 ms	1	Pre-excitation syndrome (140 ms and delta wave)
LVH (Cornell voltage criteria)	0	
LVH (Sokolow–Lyon criteria)	3	
ST deviation >0.1 mV	0	
QTc interval >440 ms	0	

### Statistical methods

We used Pearson’s correlation coefficient to compare the electrographic parameters between the two ECGs. The linear correlation of the electrocardiographic parameters was analyzed using the SPSS Statistics version 19. The correlation coefficients ranged from −1.0 to +1.0, with −1.0 and +1.0 representing a perfect negative and a perfect positive correlation, respectively. As the value approaches to 1.0, the correlation becomes stronger and a value between 0.5 and 1.0 indicates a strong correlation. The P value for the each comparison was calculated to determine the statistical significance of the results.

### Institutional Review Board

This study was approved by the Institutional Review Board at the University of Illinois at Urbana-Champaign, #12354. The new wireless electrocardiographic transmission device was also approved by the Institutional Review Board at the University of Illinois at Urbana-Champaign to be safe for testing in athletes.

## Results

### QRS duration

**Fig. 4:**
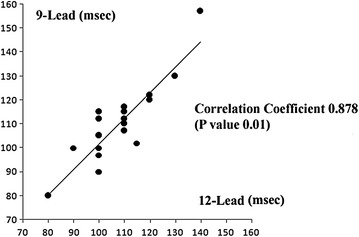
The correlation coefficient and correlation graph of each electrocardiographic parameter. The QRS duration on the 12-lead ECG (*horizontal axis*) and the 9-lead ECG (*vertical axis*).

We measured the longest QRS duration on the 12-lead and 9-lead ECGs. The correlation coefficient was calculated as 0.878 with a P value of 0.01 (Fig. 4), indicating that the QRS duration strongly correlated between the two ECGs.

### Left ventricular hypertrophy

**Fig. 5:**
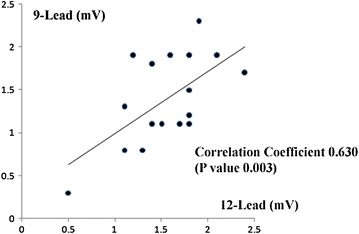
The correlation coefficient and correlation graph of each electrocardiographic parameter. The left ventricular hypertrophy using the Cornell voltage criteria on the 12-lead ECG (*horizontal axis*) and the 9-lead ECG (*vertical axis*).

**Fig. 6:**
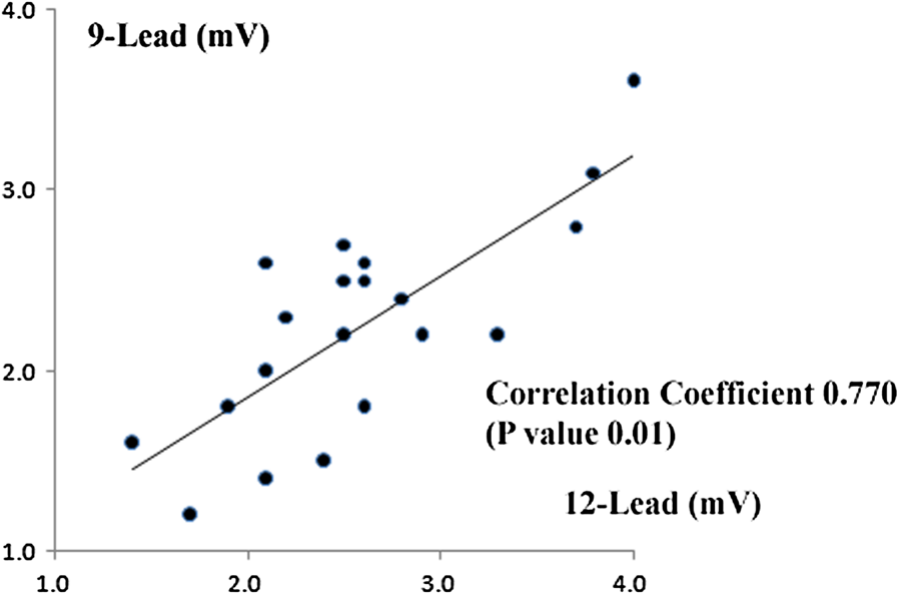
The correlation coefficient and correlation graph of each electrocardiographic parameter. The left ventricular hypertrophy using the Sokolow–Lyon criteria on the 12-lead ECG (*horizontal axis*) and the 9-lead ECG (*vertical axis*).

We compared LVH using the Cornell voltage and Sokolow–Lyon criteria on the two ECGs. The correlation coefficient was measured as 0.630 with a P value of 0.003 for the Cornell voltage criteria (Fig. 5). The correlation coefficient was 0.770 with a P value of 0.01 for the Sokolow–Lyon criteria (Fig. 6). Both of the comparisons showed strong correlation between the two ECGs to detect LVH.

### ST deviation

**Fig. 7:**
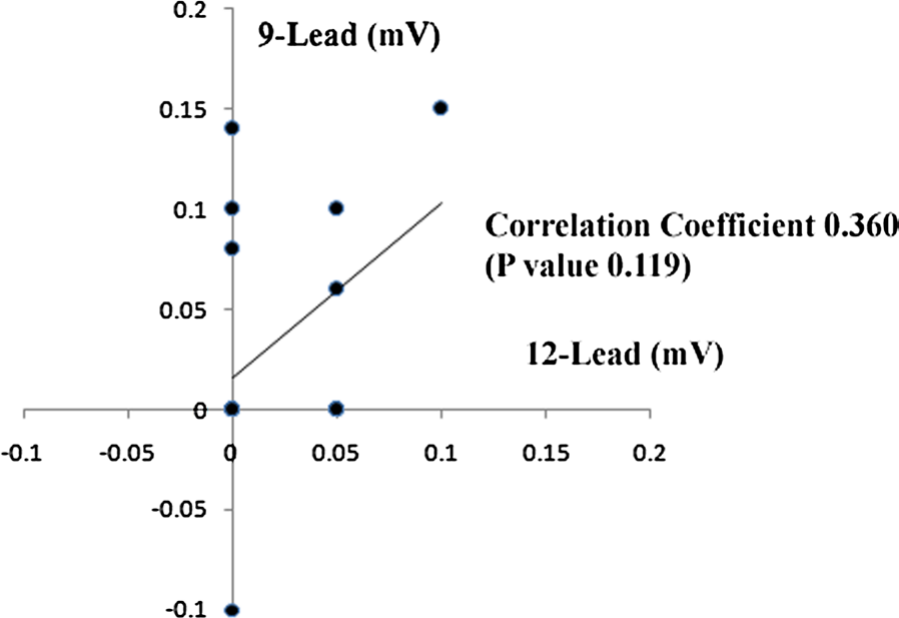
The correlation coefficient and correlation graph of each electrocardiographic parameter. The ST deviation in lead V1 on the 12-lead ECG (*horizontal axis*) and the 9-lead ECG (*vertical axis*).

We compared the ST deviation of lead V1 on the two ECGs. The correlation coefficient was calculated as 0.360 with a P value of 0.119 (Fig. 7). The correlation was weak without the statistical significance. On most of the ECGs, there was no appreciable ST deviation.

### QT prolongation

**Fig. 8:**
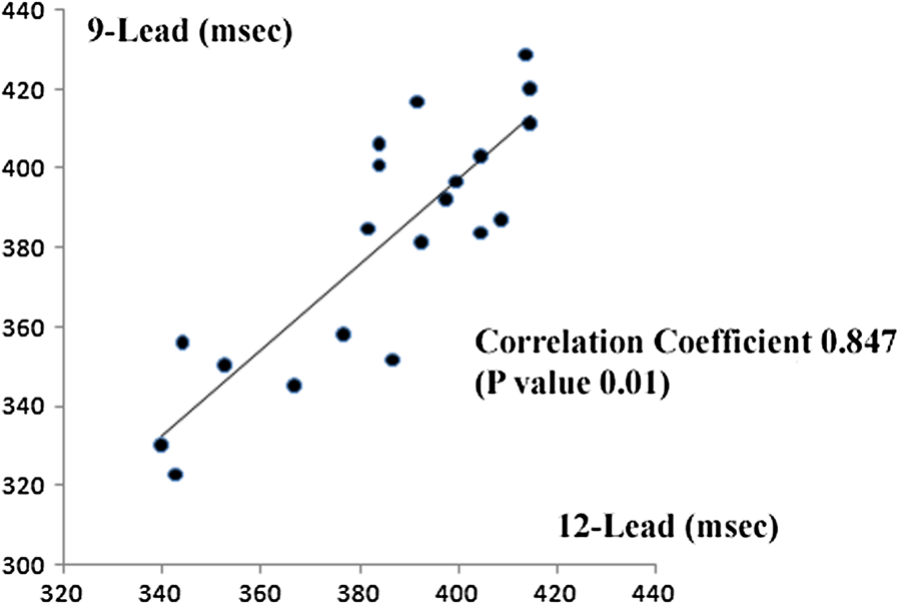
The correlation coefficient and correlation graph of each electrocardiographic parameter. The corrected QT interval on the 12-lead ECG (*horizontal axis*) and the 9-lead ECG (*vertical axis*).

We compared the corrected QT intervals between the two ECGs to screen for long QT and short QT syndrome. We calculated the corrected QT intervals by dividing the QT interval by the root square of the RR interval. The correlation coefficient was 0.847 with a P value of 0.01 (Fig. 8), indicating that the correlation is significantly positive.

## Discussion

The prevention of sudden cardiac death in young competitive athletes with a conventional 12-lead ECG continues to be controversial. We applied our newly-developed 9-lead wireless electrocardiographic transmission device to obtain equivalent information at lower cost. Our new device has 9 leads and wirelessly transmits the data to a secure server, which could pave the way to rendering ECG-based screening inexpensive and widely available. This was a pilot study to validate the usefulness of our new electrocardiographic device by comparing various electrocardiographic parameters between the two ECGs. Our new 9-lead ECG was comparable to the conventional 12-lead ECG in detecting widened QRS complexes, LVH and corrected QT abnormalities. A widened QRS complex can be used to screen for pre-excitation syndromes, arrhythmogenic right ventricular cardiomyopathy and bundle branch blocks. The Cornell voltage or Sokolow–Lyon criteria can be used to screen for LVH and hypertrophic cardiomyopathy. Prolonged or shortened corrected QT intervals can be used to detect long QT or short QT syndrome.

The most common cause of sudden cardiac death in young competitive athletes in the United States is hypertrophic cardiomyopathy (26.4 %), followed by commotio cordis (19.9 %), coronary artery anomalies (13.7 %), LVH of indeterminate cause (7.5 %), myocarditis (5.2 %), ruptured aortic aneurysm (3.1 %), arrhythmogenic right ventricular cardiomyopathy (2.8 %), bridged coronary artery (2.8 %), aortic valve stenosis (2.6 %), atherosclerotic coronary artery disease (2.6 %), dilated cardiomyopathy (2.3 %), myxomatous mitral valve degeneration (2.3 %), asthma (2.1 %), heat stroke (1.6 %), drug abuse (1.0 %), other cardiovascular causes (1.0 %) and long QT syndrome (0.8 %) [2]. Among these causes, only hypertrophic cardiomyopathy, LVH, arrhythmogenic right ventricular cardiomyopathy and long QT syndrome can be screened using a conventional 12-lead ECG. Our study demonstrated that the new 9-lead ECG is equivalent to the 12-lead ECG in determining these parameters.

There are a few limitations to our study. First, our device is not yet commercially available and thus we cannot determine how much it will cost. However, because our device is simple, small and records using only 9 leads, it would be much less expensive than conventional electrocardiographic machines. When we have the cost of our device determined, we will analyze the cost-effectiveness of our device. Second, we included only a small cohort of athletes, which might not be sufficient to validate these ECG results for the overall athlete population. This is a pilot study and we are planning to include a larger number of athletes in baseball, basketball, football and ice hockey. We are also planning to check LVH with echocardiogram to determine how well our wireless transmission device predicts LVH. We are planning to broaden our study to check exercise-induced ECG changes along with heart rate variability and signal-averaged ECG. We need more testing to ensure that our device can provide us the same quality of ECGs while athletes are doing activities of daily living or even exercising. Third, the ST deviation in lead V1 only showed a weak correlation between the two ECGs. We know that the ST deviation in lead V1 varies depending on the lead placement and the causes of sudden cardiac death which can be detected by ST deviation in lead V1 are only Brugada syndrome and arrhythmogenic right ventricular cardiomyopahty. Brugada syndrome is a rare cause of sudden cardiac death and arrhythmogenic right ventricular cardiomyopathy only comprises 2.8 % of sudden cardiac death in the United States. Thus this weak correlation of ST deviation in lead V1 will not affect the overall validity of our device.

The new 9-lead wireless electrocardiographic transmission device is still under investigation and modification. Our device is very convenient to use and apply. We are planning to apply our device while the athletes are doing activities of daily living and even exercising in the field. This convenience of testing will be another advantage of having wireless transmission system of our device. The measurement of the various electrocardiographic parameters is easy to perform with zooming and panning functions of the digital ruler. Cloud computing will allow us to create population norms and to compare ECGs for the same athlete. A significant value would trigger a further analysis by a physician and result in further work-up such as echocardiogram. Because only a small number of ECGs would need a physician reading, this device could be potentially less expensive than conventional 12-lead ECG. We hope that this new device will improve the cost-effectiveness of electrocardiographic screening for the prevention of sudden cardiac death in young competitive athletes.

## Conclusions

Our newly developed wireless 9-lead electrocardiogram demonstrated significant correlations with a conventional 12-lead electrocardiogram in terms of QRS duration, left ventricular hypertrophy and corrected QT interval.

## Abbreviations

ECG: electrocardiogram
ESC: European Society of Cardiology
AHA: American Heart Association
ACC: American College of Cardiology
LVH: left ventricular hypertrophy
